# Walking Attenuates Postprandial Glycemic Response: What Else Can We Do without Leaving Home or the Office?

**DOI:** 10.3390/ijerph20010253

**Published:** 2022-12-24

**Authors:** Alessio Bellini, Andrea Nicolò, Jacopo Emanuele Rocchi, Ilenia Bazzucchi, Massimo Sacchetti

**Affiliations:** Department of Movement, Human and Health Sciences, University of Rome “Foro Italico”, Piazza Lauro De Bosis 6, 00135 Rome, Italy

**Keywords:** postprandial exercise, post-meal exercise, post-meal glucose, exercise strategies, walking, neuromuscular electrical stimulation, indoor exercise, breakfast, morning

## Abstract

We evaluated the effects of different exercise types suitable for a home/work setting on the postprandial glucose response. Twenty-three healthy, active, young individuals performed one of two studies (12 in Study 1 and 11 in Study 2), with four randomized protocols each. After a meal high in carbohydrate content (1 g of carbohydrate per kg of body weight), in Study 1, participants performed 30 min of either walking (WALK), bench stepping exercise (STEP) or isometric wall squat (SQUAT); in Study 2, participants performed 30 min of either walking (WALK), neuromuscular electrical stimulation alone (P_NMES) or superimposed on voluntary muscle contraction (VC_NMES). In both studies, participants performed a prolonged sitting condition (CON) that was compared to the exercise sessions. In Study 1, WALK and STEP significantly reduced the glucose peak compared to CON (*p* < 0.011). In Study 2, the peak was significantly reduced in WALK compared to CON, P_NMES and VC_NMES (*p* < 0.011) and in VC_NMES compared to CON and P_NMES (*p* < 0.011). A significant reduction of 3 h glucose iAUC was found for WALK and VC_NMES compared to CON and P_NMES (*p* < 0.033). In conclusion, WALK is the most effective strategy for improving the postprandial glycemic response. However, STEP and VC_NMES can also be used for reducing postprandial glycemia.

## 1. Introduction

Exercise is a valuable tool for improving the acute glycemic response to a meal in healthy and diabetic individuals. Several studies have suggested that exercise-induced reductions in postprandial glycemia have important effects on the cardiometabolic state of individuals [1,2,3,4,5,6,7,8]. The effect of exercise on the glycemic response may change considerably depending on when an exercise session is performed with respect to the meal. For instance, postprandial exercise is more effective than preprandial exercise [1,2,5,6,9,10,11], especially when started before the glycemic peak is reached [2,11,12]. The timing of exercise with respect to the meal is one of the most important parameters to consider when prescribing exercise for improving the postprandial glycemic response. Hence, the optimization of the post-meal glucose control through exercise should take into account the temporal distance between exercise and the meal. Likewise, exercise prescription should consider other parameters that may differently affect the glycemic response to a meal.

Unlike timing manipulation, variations in exercise intensity and duration affect the postprandial glycemic response to a limited extent [2,13,14,15]. Likewise, different exercise types (e.g., running, walking, cycling, resistance exercise and different combinations of resistance and aerobic exercise) are similarly effective in reducing the postprandial glucose response [2,4,5], with only minor between-type differences observed in most of the cases. Therefore, it has been suggested that 30 min of brisk walking is a valid exercise strategy for different populations [2,3]. However, the fact that different postprandial exercise types have similar effects on the glycemic response has other implications that require further consideration.

Despite the well-established effectiveness of postprandial exercise, adherence to this practice is often insufficient, as for exercise in general [16,17,18,19,20]. However, postprandial exercise adherence has additional challenges, especially considering that its effectiveness is reduced if exercise is not performed soon (i.e., <30 min) after the meal [2,3,11,12]. One of these challenges is the location where the exercise is performed. For instance, while walking may be suitable for a variety of situations, a large number of meals are consumed either at home or in the workplace, where walking may not always be a suitable strategy, depending on space restrictions, environmental conditions, individual preference and other factors. Therefore, it is particularly relevant to evaluate the effectiveness of different exercise strategies that can be performed in home/workplace settings, but little research has been done in this area.

Alternative exercise strategies suitable for home/workplace settings may, for instance, require minimal equipment or small spaces to be performed (e.g., bench stepping and isometric exercise). Other interesting exercise modalities are those requiring minimal cognitive or physical effort, which are among the main barriers to exercise adherence [21,22,23]. As such, a potentially promising solution is neuromuscular electrical stimulation (NMES), as it requires minimal to moderate collaboration and can even be performed while sitting or lying down. The feasibility of the different options for exercising in less conventional settings should then be evaluated in relation to the need to exercise in close proximity to the meal, as well as in relation to its effectiveness in attenuating postprandial blood glucose. Some attempts have been made to test the effectiveness of NMES on the glycemic response to a meal based on the premise that at least part of the effect of exercise depends on muscle contraction, but the findings obtained so far are controversial and limited [24,25,26]. More studies are required in order to clarify whether NMES is an effective strategy for improving the post-meal glucose response in different populations.

This investigation is composed of two different studies collectively aiming to evaluate the effectiveness of different postprandial exercise strategies on the glycemic response to a standard mixed meal. Exercise strategies requiring little equipment, space or effort were tested (i.e., bench stepping exercise, isometric wall squat, passive NMES and NMES superimposed to voluntary muscle contractions) and compared with a session of 30-min brisk walking. This investigation has potential implications for the implementation of effective postprandial exercise strategies in home/workplace settings.

## 2. Materials and Methods

### 2.1. Participants

Twenty-five healthy, active (meeting the World Health Organization physical activity guidelines [27]) young adults (20–35 years old) voluntarily participated in one of two studies. In total, twenty-three individuals completed all the trials of the study, while two abandoned the study for personal reasons. All participants were students from local universities recruited through word of mouth. Participants presenting cardiometabolic disorders or assuming pharmacological treatments were excluded from the study. Detailed information on participants is given in Table 1. The studies were conducted in accordance with the Declaration of Helsinki, and ethical approval for this study was granted by the Local Ethical Committee (CAR 102/2021).

### 2.2. Overview

**Figure 1:**
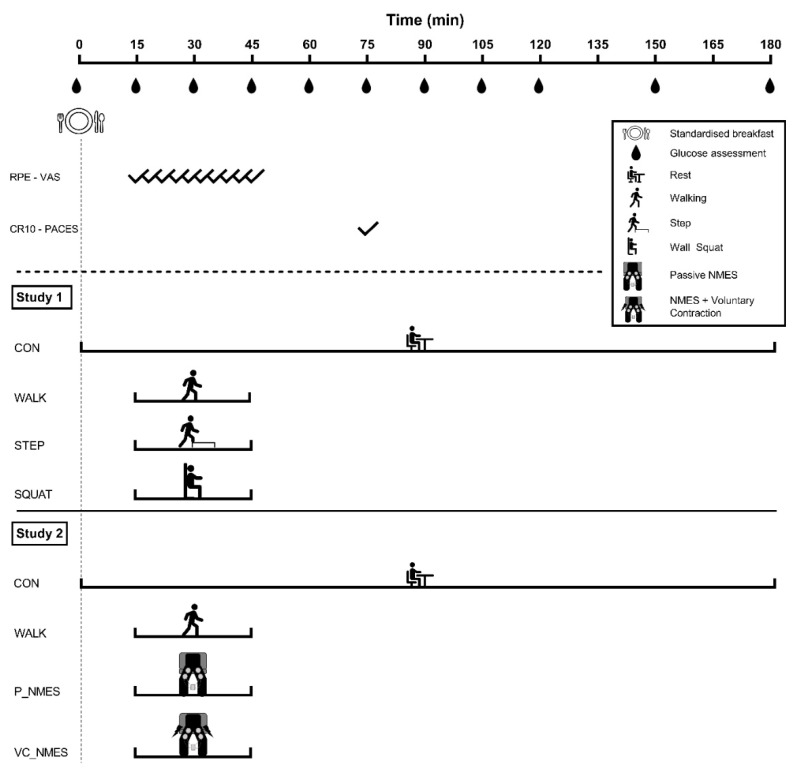
Schematic representation of the two studies. Participants consumed a meal consisting of 1 g of CHO per kg of body weight at the beginning of each visit in both studies. In Study 1, participants performed 30 min of walking (WALK), bench stepping (STEP) or isometric wall squat (SQUAT). In Study 2, participants performed 30 min of walking (WALK), passive neuromuscular electrical stimulation (P_NMES), or NMES superimposed on voluntary muscle contraction (VC_NMES). After the 30 min of exercise, participants remained seated for the remaining experimental period. The exercise sessions in both studies were compared to a control condition (CON), during which participants of each study remained seated for the whole experimental period. Every 3 min during the exercise sessions, the rate of perceived exertion (RPE) and the visual analogue scale (VAS) for discomfort were evaluated. At the end of the exercise, the Category Ratio 10 (CR10) and the Physical Activity Enjoyment Scale questionnaire (PACES) were also used to assess perceived exertion and enjoyment of the exercise session. Glucose was regularly measured throughout the experimental period.

One of two repeated measures crossover studies was performed by participants. Each of the two studies compared three different exercise conditions with a control condition, as graphically shown in Figure 1. At the beginning of each study, a familiarization session was performed, during which they were informed about all the experimental procedures adopted during the four experimental visits. Each study consisted of four experimental visits, performed in a randomized order and lasting 3 h each. In order to avoid any residual effect of exercise, 7 ± 4 days (Study 1) and 10 ± 2 days (Study 2) of rest were maintained between visits [28]. For both studies, at the beginning of each visit, participants consumed a standardized breakfast high in carbohydrate (CHO) content. After the meal, in each study, participants performed 30 min of exercise or remained seated for the whole experimental visit (CON), as detailed below.

### 2.3. Pre-session Procedures

All participants attended a familiarization session, during which all the experimental protocols and procedures were presented. Furthermore, during this visit, anthropometric measures were evaluated. In addition, participants were required to abstain from moderate-to-vigorous physical activity for the 48 h before each experimental visit and to abstain from caffeine and alcohol consumption since the evening before the session. The activities performed during the 48 h and the food consumed during the 24 h before the first experimental visit were self-reported using diaries and replicated before the remaining visits.

### 2.4. Meal Consumption

In each visit, at 09.00 a.m., a standardized breakfast high in CHO content (1 g of CHO per kg of body weight) was consumed by participants. Specifically, the meal consisted of partially skimmed milk, cornflakes and sugar. In the case of lactose intolerance (4 individuals in Study 1 and 1 in Study 2), participants consumed sugared tea, rusks and jam, providing the same amount of CHO (i.e., 1 g of CHO per kg of body weight). Table 2 summarizes the energy and macronutrient content of the meal for both studies.

### 2.5. Study 1—Experimental Conditions

Twelve individuals completed all the visits of this study (Table 1). Participants were required to attend the laboratory after an overnight fast (>10 h of fasting). After the consumption of the meal, participants remained seated for the whole experimental session (CON) or performed 30 min of exercise starting 15 min after the beginning of the meal. Specifically, exercise consisted of walking (WALK), isometric wall squat (SQUAT), or bench stepping exercise (STEP).

Walking was performed at 120 steps per minute on a 50-m indoor track, rhythmically established by a digital metronome (Soundbrenner, Berlin, Germany), as previously performed [2,3]. This step cadence has been previously identified as moderate-intensity in 21–40 years old healthy adults [29,30]. The average walking speed was also calculated (1.59 ± 0.19 m·s^−1^).

SQUAT and STEP were performed with 12 cycles of 30 s of exercise followed by 60 s of rest, timed by the operators using a stopwatch. The total exercise volume was 10 min. The isometric wall squat exercise was performed with the thighs parallel to the ground and maintaining the upper limbs parallel to the trunk. STEP was performed on a step of 20 cm height. Participants were required to climb and descend the step at a step rate of 120 steps per minute, established by using a digital metronome.

During the CON session, participants were asked to remain seated and limit their movement. They were allowed to read or use the computer while sitting, and they were required to replicate the activities performed during the first visit during the other three sessions.

### 2.6. Study 2—Experimental Conditions

Twelve individuals completed all the visits of this study (Table 1). As for Study 1, participants were required to attend the laboratory after overnight fasting (>10 h of fasting). After the consumption of the meal (09.00 a.m.), participants remained seated for the whole experimental session (CON) or performed 30 min of exercise starting 15 min after the beginning of the meal. Specifically, 30 min of walking (WALK), passive NMES (P_NMES), or NMES superimposed on voluntary muscle contraction (VC_NMES) were performed.

The CON and the WALK sessions were performed with the same modalities as those previously reported in Study 1. As for Study 1, walking speed was calculated during the WALK condition (1.58 ± 0.16 m·s^−1^).

NMES was applied for 30 min in the two NMES sessions (i.e., P_NMES and VC_NMES). The stimulation was performed at a 30 Hz frequency using the Chattanooga Wireless Professional 4 Channels (DJO Global, Texas), which produced a rectangular, balanced biphasic pulse. NMES duty cycle was 30 ON s followed by 60 OFF, and it was timed by the operators using a stopwatch. During both NMES conditions, participants were seated with the knee at 90° of flexion and the feet on the floor. In the P_NMES condition, participants remained seated with no voluntary muscle activation during the stimulation period. In the VC_NMES condition, participants were asked to voluntarily contract the lower limb muscles during the 30-s stimulation period by pushing their feet on the floor. For both conditions, four self-adhesive electrodes (Compex Dura-Stick plus 5 × 10 cm) with positive polarity were placed between the muscle bellies of Vastus Medialis and Vastus lateralis for knee extensors and between the muscle bellies of Biceps femoris and Semitendinosus for knee flexors. In particular, for quadriceps, the proximal electrode was placed 5 cm below the inguinal fold on the rectus femoris line, while the distal electrode was placed 5 cm above the superior edge of the patella, following the projection of the upper electrode. For hamstrings, the proximal electrode was placed 5 cm below the gluteal sulcus, and the distal electrode was placed 5 cm above the distal tendon of the semitendinosus following the projection of the upper electrode. Both the right and left lower limbs were simultaneously stimulated. The stimulation intensity was adjusted every 5 min based on the perception of participants to ensure tolerability of the stimulation.

All of the exercise modalities tested in Studies 1 and 2 primarily involved leg muscles, as prospectively, the use of these types of exercise by the diabetic population as well could provide greater benefit by virtue of the heterogeneity of the disruption of metabolic and neuromuscular function between the lower and upper limb muscles in these patients [31,32,33,34,35].

### 2.7. Glycemic Assessment

Capillary blood glucose measures were collected and assessed by using reactive strips and glucose monitors (Contour^®^Next, Bayer HealthCare S.p.A., Milan, Italy), previously validated [36,37]. Two measures were performed at each time point, and the average of these was considered. In case of a difference greater than 10% between the two measures, a third measure was performed. As schematically shown in Figure 1, glycemic measures were collected immediately before the meal, every 15 min after the meal consumption for the first two hours, during which glycemic concentration in the blood is higher than baseline levels, and thus it requires more frequent measurements in order to evaluate the post-meal fluctuations; during the third hour, when glycemia has been previously shown to return to baseline levels, it was measured every 30 min [38]. Participants measured glycemia while sitting, and before each measure, they were requested to wash their hands to avoid possible alterations due to external factors.

### 2.8. Exercise-Related Measures

The perceived exertion was measured every 3 min at the end of the 30 s exercise bout by using the rate of perceived exertion (RPE) 6–20 Borg’s scale, while the perceived discomfort was assessed by using a 0–10 cm visual analogue scale (VAS) only during the NMES sessions (i.e., P_NMES and VC_NMES). The session rating of perceived exertion and the enjoyment of the exercise session were evaluated 30 min after the end of the exercise using the category ratio 10 (CR10) and the Physical Activity Enjoyment Scale (PACES) questionnaires, respectively.

### 2.9. Statistical Analysis

The software IBM SPSS statistics version 23.0 (SPSS Inc., Chicago, IL) was used for performing the statistical analysis. The same statistical analyses were used in both studies for all variables. The Shapiro–Wilk test was used to evaluate the normality of data distribution. A two-way repeated-measures ANOVA (condition × time) was performed for the comparison of the glycemic time course across conditions. When significant interactions were found, a one-way repeated-measures ANOVA was used to verify the simple main effect of the condition at each time point. The time-averaged positive incremental area under the curve (iAUC) was calculated at 0–60, 0–120 and 0–180 min [39] and differences between conditions were analyzed using a one-way repeated measures ANOVA. Differences between conditions of the mean blood glucose concentration at 0–180 min were evaluated using a one-way repeated measures ANOVA.

A two-way repeated measures ANOVA was used to analyze differences between conditions for the 6–20 RPE Borg scale and the VAS scores, while a one-way repeated measures ANOVA was used for the CR10 and the PACES total scores.

Mauchly’s test was performed for the evaluation of the test sphericity. When the sphericity was not assumed, the Greenhouse–Geisser or the Huynh–Feldt corrections were used for adjusting the degrees of freedom of the within-subject comparisons for ε < 0.75 and ε > 0.75, respectively. In case of significant differences, the least significant differences (LSD) correction was used for the analysis of multiple comparisons. For all statistical tests, the level of significance was set at 0.05. Partial eta squared (*η_p_*^2^) effect sizes were determined, considering *η_p_*^2^ ≥ 0.01 as small, *η_p_*^2^ ≥ 0.059 as a medium, and *η_p_*^2^ ≥ 0.138 as large [40]. Values are reported as mean ± SD in Tables and in the text and as mean (± SEM) in Figures.

## 3. Results

### 3.1. Study 1

**Figure 2:**
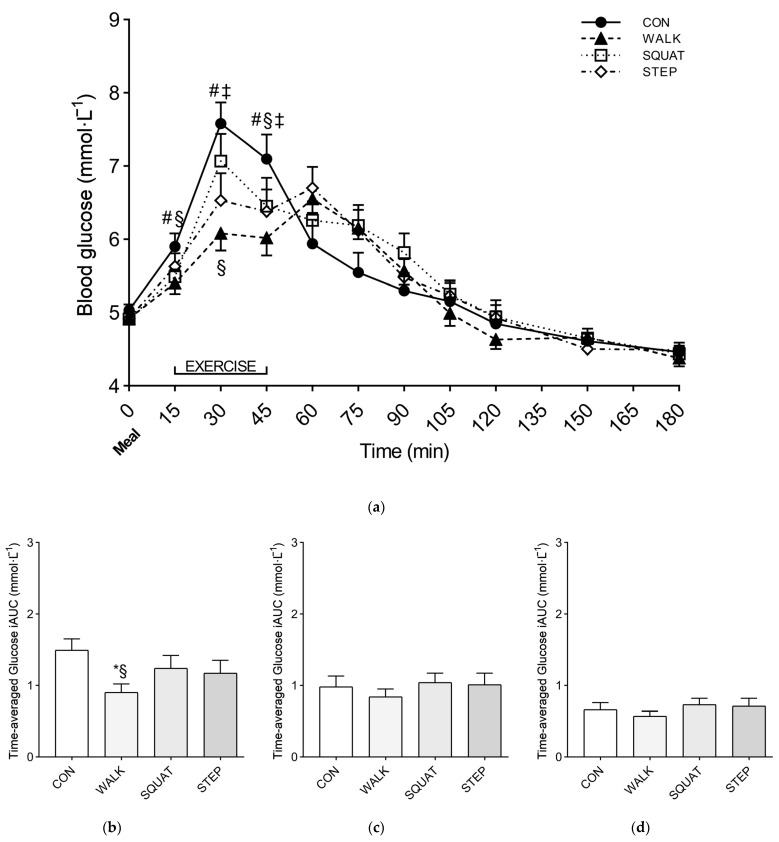
Glucose response over time (**a**) and time-averaged glucose iAUC at 0–60 h (**b**), 0–120 h (**c**), 0–180 h, and (**d**) after the meal, for Study 1. Symbols: *, *p* < 0.05 vs. CON; #, *p* < 0.05 vs. WALK; §, *p* < 0.05 vs. SQUAT; ‡, *p* < 0.05 vs. STEP. Values are reported as mean (± SEM).

A significant interaction (condition × time) (*p* < 0.001, *η_p_*^2^ = 0.315) was found when comparing the glycemic time course between conditions. At 0 min, before the meal, all the conditions showed similar glucose levels (CON, 5.03 ± 0.29 mmol·L*^−^*^1^; WALK, 4.92 ± 0.27 mmol·L*^−^*^1^; SQUAT, 4.91 ± 0.25 mmol·L*^−^*^1^; STEP, 4.92 ± 0.39 mmol·L*^−^*^1^). At 30 min, a significant reduction of the glucose peak was found for WALK (6.08 ± 0.81 mmol·L*^−^*^1^) and STEP (6.53 ± 1.27 mmol·L*^−^*^1^) compared to CON (7.58 ± 1.01 mmol·L*^−^*^1^) (*p* < 0.011), and for WALK compared with SQUAT (7.07 ± 1.27 mmol·L*^−^*^1^) (*p* = 0.005). Additional information on the simple main effect of condition at each time point is reported in Figure 2.

Significant differences between conditions were found for the time-averaged positive iAUC at 0–60 min (*p* = 0.027, *η_p_*^2^ = 0.278). Specifically, significant reductions of the iAUC were found for WALK (0.90 ± 0.43 mmol·L*^−^*^1^) compared with CON (1.49 ± 0.55 mmol·L*^−^*^1^) and SQUAT (1.24 ± 0.63 mmol·L*^−^*^1^) (*p* < 0.001 and *p* = 0.046, respectively). Conversely, no significant differences between conditions were found for iAUC at 0–120 min and 0–180 min (Figure 2).

**Figure 3:**
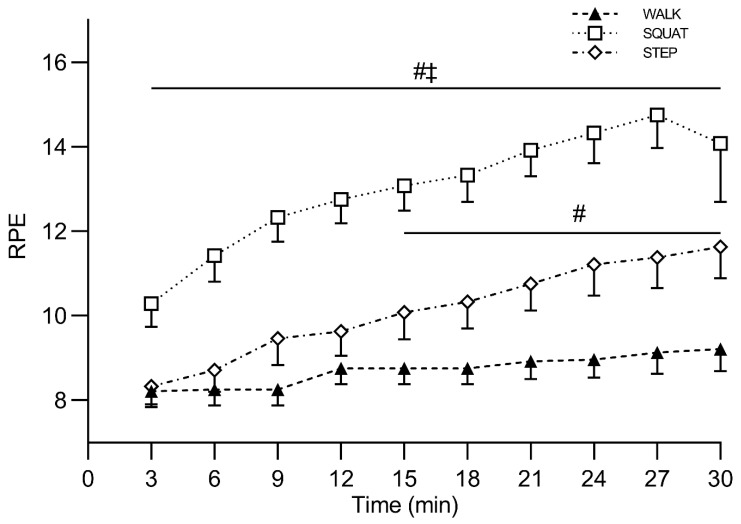
Rate of perceived exertion (6–20 RPE) during exercise, for Study 1. Symbols: #, *p* < 0.05 vs. WALK; ‡, *p* < 0.05 vs. STEP. Values are reported as mean (± SEM).

A significant interaction (condition × time) (*p* < 0.001, *η_p_*^2^ = 0.632) was found when comparing the 6–20 RPE scores between conditions. Additional information on the simple main effect of condition at each time point is reported in Figure 3. RPE was higher for SQUAT (4.83 ± 1.61) compared with WALK (2.38 ± 0.64; *p* < 0.001) and STEP (2.92 ± 0.97; *p* = 0.001). In addition, higher RPE values were found for STEP compared with WALK (*p* = 0.030). Conversely, no between-condition differences were observed for the PACES questionnaire total scores (WALK, 92.58 ± 12.20; SQUAT, 83.75 ± 19.49; STEP, 88.08 ± 17.99).

### 3.2. Study 2

**Figure 4:**
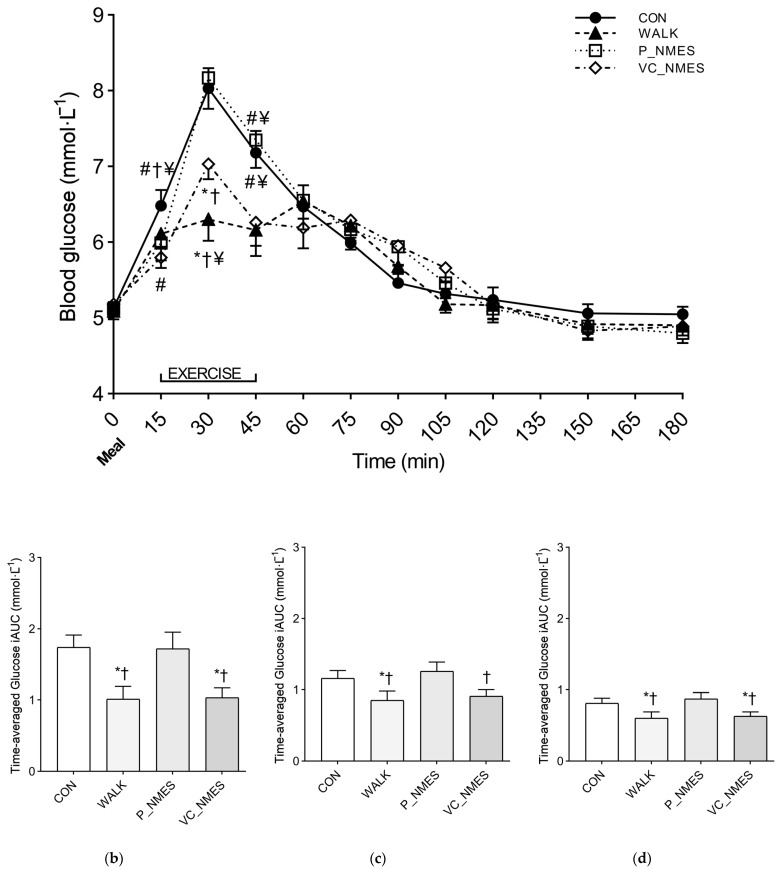
Glucose response over time (**a**) and time-averaged glucose iAUC at 0–60 h (**b**), 0–120 h (**c**), 0–180 h, and (**d**) after the meal for Study 2. Symbols: *, *p* < 0.05 vs. CON; #, *p* < 0.05 vs. WALK; †, *p* < 0.05 vs. P_NMES; ¥, *p* < 0.05 vs. VC_NMES. Values are reported as mean (± SEM).

A significant interaction (condition × time) (*p* < 0.001, *η_p_^2^* = 0.403) was found when comparing the glycemic time course between conditions. At 0 min, before the meal, all the conditions showed similar glucose levels (CON, 5.13 ± 0.28 mmol·L^−1^; WALK, 5.09 ± 0.35 mmol·L^−1^; P_NMES, 5.12 ± 0.29 mmol·L^−1^; VC_NMES, 5.18 ± 0.33 mmol·L^−1^). At 30 min, WALK (6.30 ± 0.92 mmol·L^−1^) showed a significantly reduced (*p* < 0.010) glucose peak compared with CON (8.03 ± 0.90 mmol·L^−1^), P_NMES (8.17 ± 1.35 mmol·L^−1^) and VC_NMES (7.03 ± 0.66 mmol·L^−1^). In addition, VC_NMES showed a significantly reduced (*p* < 0.010) glucose peak compared with CON and P_NMES. Additional information on the simple main effect of condition at each time point is reported in Figure 4.

Significant between-condition differences were found for the iAUC at 0–60 (*p* < 0.001, *η_p_*^2^ = 0.562), 0–120 (*p* = 0.004, *η_p_*^2^ = 0.360), and 0–180 min (*p* = 0.004, *η_p_*^2^ = 0.352). Significantly lower iAUC values were found at 0–60 (*p* < 0.003), 0–120 (*p* < 0.011), and 0–180 (*p* < 0.015) min for WALK compared with CON and P_NMES. Significantly lower iAUC values were found at 0–60 (*p* = 0.001) and 0–180 (*p* = 0.047) min for VC_NMES compared with CON. In addition, significantly lower iAUC values were found for VC_NMES at 0–60 (*p* = 0.010), 0–120 (*p* = 0.030) and 0–180 (*p* = 0.032) min compared with P_NMES (Figure 4).

**Figure 5:**
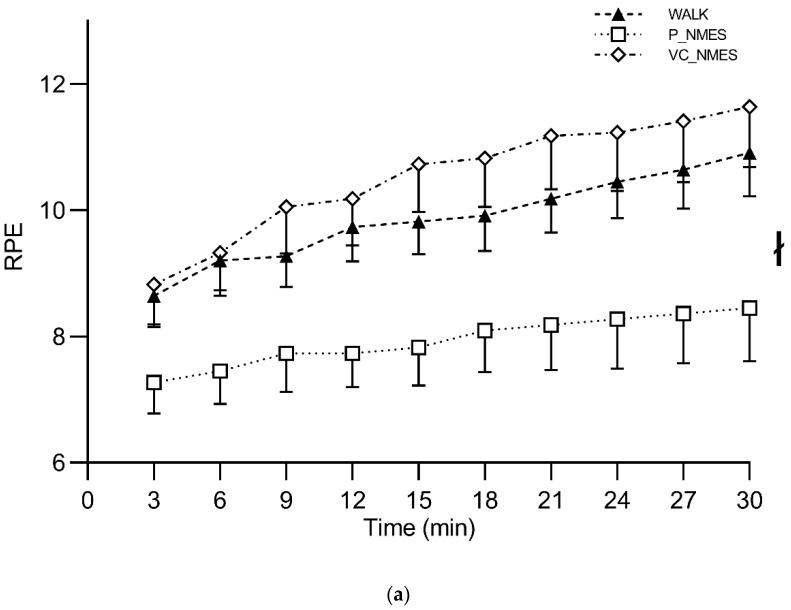

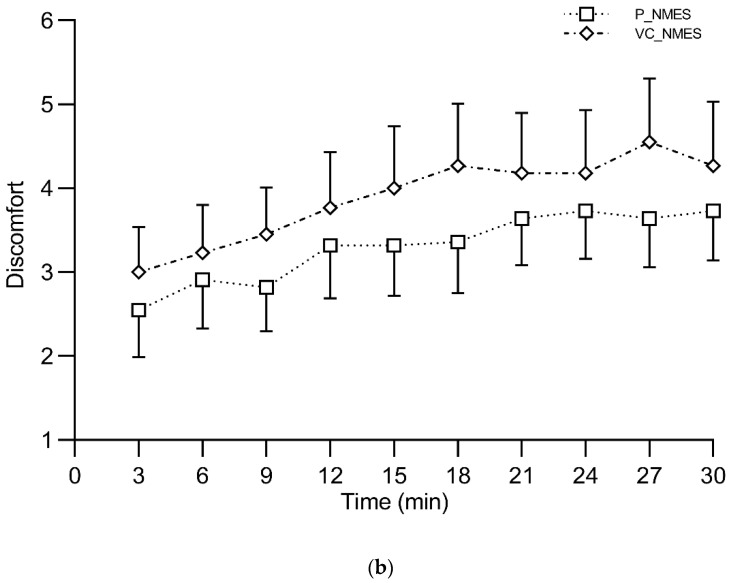
Rate of perceived exertion (6–20 RPE) (**a**) and visual analog scale (VAS) for discomfort (**b**) during exercise, for Study 2. Symbols: ł, the main effect of conditions (*p* < 0.05). Values are reported as mean (± SEM).

RPE did not show any significant main effect of time or interaction (condition × time), while a significant main effect of condition was found (*p* = 0.008, *η_p_*^2^ = 0.386). Specifically, P_NMES showed significantly lower values compared with WALK (*p* = 0.029) and VC_NMES (*p* = 0.013). A significant main effect of time (*p* < 0.001, *η_p_*^2^ = 0.413), but not of condition and interaction (condition × time), was found for the discomfort scale (Figure 5). The session rating of perceived exertion showed significantly higher scores for VC_NMES (3.45 ± 1.13) compared with WALK (2.45 ± 0.52) and P_NMES (1.77 ± 1.06) (*p* = 0.008 and *p* = 0.003, respectively). Conversely, no between-condition differences were observed for the PACES questionnaire total scores (WALK, 86.27 ± 23.72; P_NMES, 79.82 ± 18.35; VC_NMES, 79.45 ± 17.84).

## 4. Discussion

The purpose of this study was to investigate the effects of different exercise strategies suitable for home/workplace settings on postprandial glycemia. We found that (1) walking is particularly effective in improving the post-meal glucose response; (2) a reduction in the post-meal glucose peak is also achieved when performing bench stepping exercise but not isometric wall squat; and (3) NMES is only effective when combined with voluntary muscle contraction. Our findings add to those of previous studies, showing the substantial improvement of postprandial exercise on the glycemic response to meals in healthy individuals [1,2,3,5,6,8,9,10,11,12,41]. Collectively, these results have implications for planning exercise programs that improve the post-meal glycemic response and, potentially, postprandial exercise adherence.

The postprandial glucose peak showed the greatest reduction after 30 min of moderate-intensity walking started 15 min after the beginning of the meal in both studies. These findings confirm the effectiveness of this exercise type and timing in healthy individuals [2,3,42,43]. However, several barriers (e.g., lack of space) may impede people from performing postprandial walking. Based on this premise, the first study investigated the effect of bench stepping and isometric wall exercises, which require minimum space and equipment. We found that intermittent bench stepping exercise, but not wall squat, was effective for reducing the post-meal glycemic excursion. This is in line with previous findings reporting the effectiveness of 10 min of continuous stairs climbing and descending in attenuating the post-meal glucose response [44]. However, the bench stepping exercise has some advantages over stair climbing because it does not require a staircase and can be easily performed in home/work settings, with implications for exercise adherence. On the other hand, it was somewhat unexpected to observe no improvement in the postprandial glucose response in the wall-squat condition despite the same duration and timing of the exercise as the bench-stepping exercise condition. Although we did not investigate the mechanisms underlying these findings, a possible explanation may be related to a reduction of muscle blood flow that may occur during isometric exercise, which in turn may reduce muscle glucose uptake [45,46,47]. Further studies are required to investigate the factors influencing post-meal glucose control during isometric exercise.

The efficacy of bench stepping and walking exercises was not maintained for the whole postprandial period, as similar glucose iAUC levels were found for the four conditions tested over the three hours after the meal. This is probably due to the glucose rebound observed at the end of the exercise sessions, which may attenuate the benefits of exercise in the late postprandial period. A similar glucose rebound was previously observed in healthy young individuals exercising in the early postprandial period, while the rebound was counteracted when participants continued to exercise over the first three hours post-meal by performing activity breaks [2]. The somewhat similar overall glucose response of walking and bench stepping exercise is interesting in light of the different total duration of exercise (30 vs. 10 min, respectively), perceived exertion (higher during bench stepping exercise than walking), and the similar pleasure and enjoyment reported by participants. These results suggest that individuals may choose the type of exercise based on external conditions (e.g., availability of space or equipment) and individual preference with similar benefits on the glucose response to a meal.

The fact that moderate exercise is sufficient to improve the glucose response to a meal stimulates the development of exercise strategies aiming to reduce effort and/or improve adherence. Hence, in the second study, we evaluated the effects of NMES, which requires little or no cognitive and physical involvement, and is also suitable for individuals with physical impairments (e.g., injuries). We found that the passive stimulation of lower limb muscles did not elicit any improvement in the post-meal glycemic peak, showing similar values to the resting condition (i.e., CON). Similarly, performing 30 min of passive stimulation did not affect the glucose response over the three hours after the meal. These results are in contrast with previous findings that have found a reduced glucose response in diabetic and healthy sedentary individuals after 30 min of intermittent NMES [25,26]. For instance, Myiamoto et al. (2012) have shown that in old patients with type 2 diabetes, treated with oral hypoglycemic agents, 30 min of continuous passive NMES applied with a frequency of 4 Hz on the lower limb muscles reduced the glycemic response to a meal [25]. Similar results have been found in normoglycemic young sedentary individuals, in which 20 min of discontinuous (4 s ON and 12 s OFF) passive NMES at 5 Hz elicited a substantial reduction of post-meal blood glucose concentration [26]. Conversely, in our population (i.e., healthy young active individuals), passive NMES did not have any effect on the post-meal glycemic response. Different individual and stimulation protocol characteristics could underlie the differences between the present study and previous ones. In contrast to what was observed with passive NMES, we found that combining NMES with voluntary muscle contractions led to a substantial reduction of the glucose peak and of the overall glycemic response, with a similar effect to that found during the walking condition. Similar effects have been recently found in diabetic individuals; indeed, it has been reported that aerobic exercise alone elicited a similar post-meal glucose reduction when compared to whole-body NMES superimposed to resistance exercise [24]. However, in our study, participants performed muscle contractions while sitting without requiring any bodily movement. This may be particularly relevant for improving exercise adherence, especially for individuals unwilling to perform dynamic exercise after a meal or for those unable to move (e.g., in case of injuries). It is of note that a lower perceived exertion was observed after the walking session compared with the combination of NMES and voluntary contraction, despite the higher total exercise volume of the first (30 vs. 10 min, respectively). Nonetheless, participants showed similar pleasure and enjoyment when performing the two exercise modalities. Therefore, participants can choose between different exercise strategies with similar benefits in terms of post-meal glucose control based on their individual preferences and abilities.

We acknowledge that any speculation on the potential improvement in postprandial exercise adherence using the exercise strategies tested in this study should be verified with longitudinal studies. However, the present investigation addresses an important issue that facilitates the implementation of postprandial exercise in real-life scenarios, as we have evaluated the feasibility and effectiveness of different practical strategies in improving postprandial glycemia. Given the disbalance between the importance of exercise for health and limited exercise adherence, researchers are encouraged to develop and test different exercise strategies that may result in a trade-off between efficacy and suitability for the specific needs of different populations. Our findings have the potential to stimulate further research in this area.

## 5. Conclusions

We performed two studies aimed at evaluating the effects of different postprandial exercise strategies (i.e., bench stepping exercise, wall squat, walking passive NMES and NMES combined with active muscle contractions) on the glucose response to a meal in healthy individuals. We found that walking is the most effective exercise type in reducing the postprandial glucose peak among those tested. However, bench stepping exercise and NMES combined with voluntary muscle contractions substantially improved the glucose response to a meal. These findings suggest that individuals can choose among several postprandial exercise types based on their necessities, preference, and abilities, with potential implications for postprandial exercise adherence. However, our findings obtained in young, healthy individuals may not necessarily translate to individuals with dysregulated metabolism, where different pathophysiological states, comorbidities and drugs may interfere with the effects of exercise on the post-meal glucose concentration. Hence, for this reason, future studies should focus on evaluating the effects of these exercise strategies in individuals with dysmetabolism, where participation in exercise is even more problematic than in healthy individuals.

## Figures and Tables

**Table 1:** Characteristics of participants in the two studies.

	Study 1	Study 2
Sample Size (M/F)	12 (5/7)	11 (9/2)
Age (years)	24 ± 3	27 ± 4
Weight (kg)	69 ± 15	70 ± 9
Height (m)	1.69 ± 0.10	1.76 ± 0.08
BMI (kg/m^2^)	23.9 ± 2.9	22.7 ± 2.1

Abbreviations: M, male; F, female; BMI, body mass index. Data are expressed as mean ± SD.

**Table 2:** Macronutrient content and energy intake of breakfast in the two studies.

	Study 1	Study 2
Energy Intake (kcal)	353.20 ± 81.63	378.32 ± 67.66
Carbohydrate (g)	69.56 ± 14.97	69.64 ± 9.80
Protein (g)	10.02 ± 4.06	13.36 ± 4.35
Fat (g)	3.85 ± 1.45	5.16 ± 1.72
Carbohydrate (%)	79.48 ± 4.00	74.51 ± 5.16
Protein (%)	11.16 ± 3.07	13.83 ± 3.09
Fat (%)	9.67 ± 2.25	11.97 ± 2.73

Data are expressed as mean ± SD.

## Data Availability

The data presented in this study are available on request from the corresponding author.
